# High-Efficiency Chemical-Mechanical Magnetorheological Finishing for Ultra-Smooth Single-Crystal Silicon

**DOI:** 10.3390/nano13030398

**Published:** 2023-01-18

**Authors:** Zhifan Lin, Hao Hu, Yifan Dai, Yaoyu Zhong, Shuai Xue

**Affiliations:** 1 Laboratory of Science and Technology on Integrated Logistics Support, National University of Defense Technology, Changsha 410073, China; 2 College of Intelligence Science and Technology, National University of Defense Technology, Changsha 410073, China; 3 National Innovation Institute of Defense Technology, Academy of Military Sciences, Beijing 100091, China

**Keywords:** single-crystal silicon, chemical-mechanical magnetorheological finishing, ultra-smooth surface, magnetorheological finishing fluid

## Abstract

To improve the material removal efficiency and surface quality of single-crystal silicon after magnetorheological finishing, a novel green chemical-mechanical magnetorheological finishing (CMMRF) fluid was developed. The main components of the CMMRF fluid are nano-Fe_3_O_4_, H_2_O_2_, CH_3_COOH, nanodiamond, carbonyl iron powder, and deionized water. The novel CMMRF fluid can simultaneously achieve Ra 0.32 nm (0.47 mm × 0.35 mm measurement area), Ra 0.22 nm (5 μm × 5 μm measurement area), and 1.91 × 10^−2^ mm^3^/min material removal efficiency. Comprehensive studies utilizing a scanning electron microscope and a magnetic rheometer show that the CMMRF fluid has a high mechanical removal effect due to the well-dispersed nanodiamond and nano-Fe_3_O_4_ particles. The results of Fourier transform infrared spectra and Young’s modulus test reveal the mechanism of the chemical reaction and the mechanical characteristics deterioration of the modified layer. Under co-enhanced chemical and mechanical effects, an ultra-smooth and highly efficient MRF technology for single-crystal silicon is realized.

## 1. Introduction

An X-ray reflector is the essential part of the synchrotron radiation source [1,2,3]. Its manufacturing precision and surface quality have a decisive effect on the focusing of the X-ray beam [4,5,6]. X-ray reflectors are typically designed as complicated, curved surfaces. Due to the grazing incidence features of X-ray, the reflectors utilized in synchrotron radiation sources are often elongated, and their length in the direction of X-ray incidence can reach hundreds of millimeters, or even longer [7,8,9].

The preferred substrate material for X-ray reflectors is single-crystal silicon, which has exceptional thermal characteristics, mechanical properties, and processability.

Magnetorheological finishing (MRF) is a deterministic sub-aperture polishing technique based on a magnetically sensitive fluid that removes material via a shearing mechanism with minimal normal load [10,11]. Driven by a five-axis motion system, the MRF tool can improve surface quality [12,13], remove subsurface damage [14,15], and eliminate residual stress [16] when correcting surface form errors. Therefore, the MRF is frequently employed in the fine correction stages of the X-ray reflector manufacturing process [17,18].

X-ray reflectors require the roughness of the single-crystal silicon substrate to be Ra 0.3 nm or lower. Current MRF technology is still far from achieving this level, so an additional ultra-smooth process needs to be introduced to improve the surface quality after MRF [19]. In addition, the single-crystal silicon substrate after grinding may have form error ranging from submicron to several microns [20], making the polishing and surface correction cycle quite lengthy [21]. Improving the removal efficiency and the ability to process ultra-smooth surfaces of MRF is of significant benefit to the manufacture of X-ray reflectors, as well as large-aperture high-power laser optical components and ultra-large-aperture astronomical telescopes [22,23].

Numerous significant efforts have been devoted to enhancing the efficiency or surface quality of MRF. In terms of improving the efficiency of MRF, QED novel developments have been made with the fluid nozzle, magnetic field, and wheel geometry and wheel size to increase the removal rate. Custom nozzle shapes are used to create a very wide MRF ribbon resulting in a much wider removal function spot [24]. The polishing wheel with a 500 mm diameter has resulted in a significant increase in polishing speed and removal efficiency [25]. Ren substituted a belt for the large-diameter polishing wheel, which reduced the overall size of the device and increased the polishing contact area [26]. Jung sintered carbon nanotubes and iron powder as a new form of abrasive, which not only improved the durability of the abrasive, but also improved the material removal efficiency of MRF [27]. Jang incorporated the electrochemical corrosion effect into MRF for conducting hard materials; the formed oxide layer has 20% less hardness than the substrate, effectively enhancing the efficiency of MRF [28]. In terms of the ultra-smooth MRF process, Wang discovered that dual-rotor movement randomizes the material removal of MRF, prevents the formation of directional grooves on the surface, and considerably improves the surface roughness [29]. Zhang optimized the formulation of the MRF fluid and employed nanodiamonds as the abrasive for single-crystal silicon polishing, avoiding the comet tail phenomenon and the surface roughness of the aspheric single-crystal silicon after MRF achieved Ra 1.2 nm [30]. QED has reported a novel MRF fluid that can process an ultra-smooth surface of Ra 0.2 nm on glass-ceramics [31]. Sidpara developed a surface roughness prediction model based on the ratio of MRF fluid and process parameters, and optimized the process parameters to reduce the surface roughness of single-crystal silicon from Ra 1300 nm to Ra 8 nm [32]. Most studies do not simultaneously improve efficiency and surface quality. To address this challenge, it is preferable to modify the surface with external energy (chemical energy, light energy, or electric energy) and then remove the modified layer through mechanical action.

Chemical-mechanical polishing (CMP) is a process in which the material is removed by a chemical-mechanical synergetic mechanism, and it is regarded as the most effective method for achieving the sub-nano surface roughness [33,34]. Inspired by CMP technology, an eco-friendly chemical-mechanical magnetorheological finishing (CMMRF) fluid was developed. This fluid will be compatible with the principles of green and sustainable development and will not harm the environment or the operators.

With the advancement of the semiconductor industry, the CMP technology of single-crystal silicon has reached a highly developed state. In an alkaline environment, the nano-SiO_2_ abrasive can react with the surface of single-crystal silicon to generate a soft layer, which is then removed by the abrasive to reveal a smooth substrate [35]. However, compared with the wafer planarization process, the minimal normal load and the small polishing contact area of MRF cause the efficiency of MRF fluid configured with nano-SiO_2_ is quite low. To achieve a high-efficiency and ultra-smooth CMMRF process for single-crystal silicon, it is important to find alternative chemical additives and abrasives.

The objective of this study is to configure a novel eco-friendly CMMRF fluid to achieve efficient ultra-smooth processing of single-crystal silicon. The CMMRF fluid is composed of nanodiamond abrasives, carbonyl iron powder (CIP), nano-Fe_3_O_4_, H_2_O_2_, CH_3_COOH, and deionized water. The novel polishing fluid can achieve a volume removal rate of 1.91 × 10^−2^ mm^3^/min and a surface roughness of Ra 0.22 nm (5 μm × 5 μm). The roughness is reduced by 51.2% and the efficiency has grown by 196.9% in comparison to the pure nanodiamond MRF fluid. These results demonstrate that the CMMRF fluid has an excellent performance in the high-efficiency process of ultra-smooth single-crystal silicon. In order to optimize and clarify the mechanism of the novel CMMRF fluid, a series of characterization experiments were carried out. The magnetic rheometer results demonstrate that nano-Fe_3_O_4_ increases the shear yield strength of MRF fluid, and the nanodiamond abrasives with the sharp cutting-edge lead to such a high material removal rate. A heterogeneous Fenton reagent composed of H_2_O_2_, nano-Fe_3_O_4_, and CH_3_COOH was characterized and optimized by spectrophotometer. The heterogeneous Fenton reagents generates a large number of hydroxyl radicals(·OH) which oxidize the surface and generate a silicon dioxide modified layer. The Young’s modulus test and Fourier transform infrared (FTIR) spectra indicate that the hydroxyl group in the solution can enhance the hydrolysis reaction of the modified layer, destroy its network structure, and degrade its mechanical properties. Compared to the mechanical MRF fluid, the novel CMMRF fluid considerably improves chemical and mechanical effects, enabling a high-efficiency ultra-smooth single-crystal silicon process.

## 2. Samples Preparation and Characterizations

The materials used to configure the MRF fluid in this paper include CIP with an average particle size of 2 μm, Fe_3_O_4_ with an average particle size of 20 nm, diamond polishing particles with an average particle size of 150 nm, deionized water, CH_3_COOH, PEG200, HCl, and NaOH. The details of different constituents used for the MRF are mentioned in Table 1.

**Figure 1:**
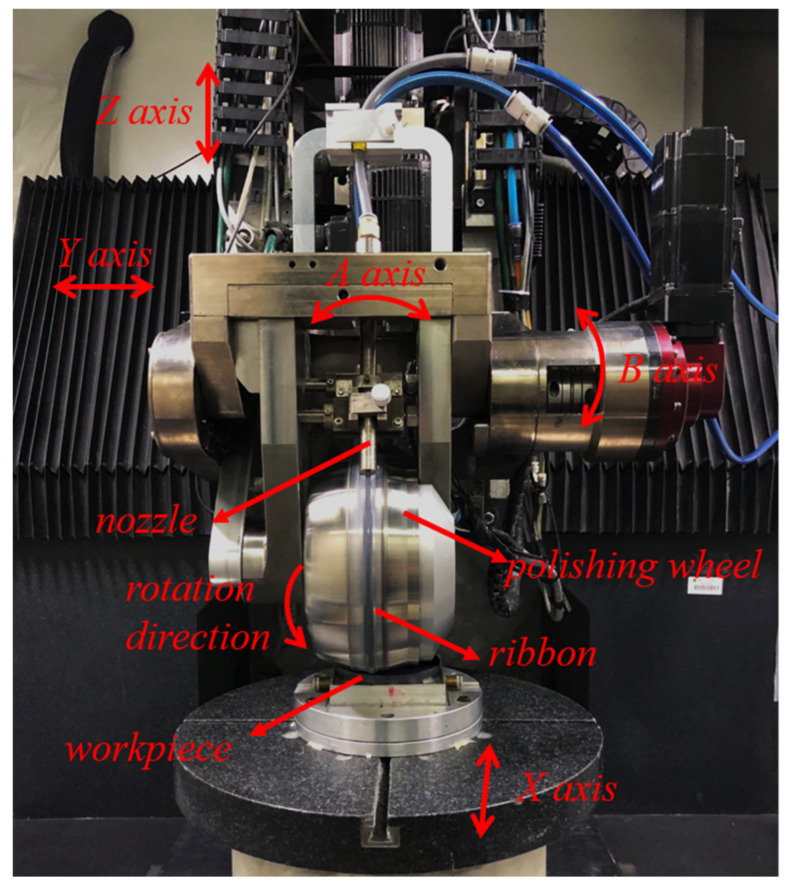
The self-developed MRF machine tool.

Single-crystal silicon CZ-(111), with a diameter of 100 mm and a thickness of 10 mm, was utilized as the experiment sample for roughness and material removal efficiency. The initial surface roughness was approximately Ra 1 nm after fully pitched polishing, and the form error PV was less than 1 μm. As shown in Figure 1, the self-developed MRF machine tool (KDUPF650-7, NUDT, Changsha, China) was utilized to process the single-crystal silicon. The electromagnet was installed inside the polishing wheel, and the intensity of the magnetic field was controlled by adjusting the current of the electromagnet coil. The polishing wheel has a diameter of 200 mm and is driven by a motor. The magnetorheological fluid is stored in the storage tank, flows out from the nozzle through the centrifugal pump, and is recovered to realize the circulation. The process parameters are as follows: the magnetic field current is 8A; the polishing wheel speed is 180 rpm; the flow rate is 200 L/h; and the ribbon penetration depth is 0.3 mm. The surface roughness results are obtained by MRF uniformly polishing. The uniform polishing path is a raster path with a step of 1 mm, and the feed rate of the MRF tool is 100 mm/min. The uniform polishing runs once on each sample. The MRF tool influence function (TIF) was obtained from the fixed-point experiment, and the volume removal rate (VRR) was calculated according to the TIF.

The sample roughness results were measured by an atomic force microscope (AFM, Dimension icon, Bruker, Billerica, MA, USA) and a white light interferometer (WLI, New view 7000, Zygo, Middleboro, MA, USA). The measuring area of the AFM is 5 μm × 5 μm, and the tapping mode was used for surface microscopic topography imaging. The WLI has a magnification of 20 times, and the field of view is 0.47 mm × 0.35 mm.

The content of ·OH in CMMRF fluid determines the oxidation effect of single-crystal silicon. Spectrophotometry was used to measure the content of ·OH in the CMMRF fluid. 2,3-dihydroxybenzoic acid and 2,5-dihydroxybenzoic acid produced by the reaction of salicylic acid with ·OH will have strong absorption peaks at 510 nm. We prepared a mixed solution of salicylic acid, ethanol, and deionized water as a catcher for ·OH, in which the concentration of salicylic acid was 1.8 mol/L, and the ratio of ethanol to water was 1:3. A UV-VIS-NIR 3600(Shimadzu, Kyoto, Japan) spectrophotometer was used to measure the absorbance of mixed solutions containing different formulas of CMMRF fluid and catcher.

MRF base fluid refers to the combination of the liquid components in the MRF fluid. In the CMMRF base fluid environment, the dispersion condition of diamond abrasive and CIP with nano-Fe_3_O_4_ was photographed using a scanning electron microscope (SEM, MIRA3 AMU, TESCAN, Brno, Czech Republic). The shear yield strength of the MRF fluid was measured by Anton Paar MCR302 rheometer with an MRD180 module.

To characterize the chemical-mechanical mechanism of CMMRF fluid on single-crystal silicon, Young’s modulus and FTIR tests were conducted on single-crystal silicon CZ- (111) samples with a diameter of 10 mm and a thickness of 3 mm. After fully pitched polishing, the samples’ surface roughness was approximately Ra 1 nm. Using a plasma processing system (Ion wave 10), we conducted 1000 W power Ar gas plasma cleaning for 60 s to eliminate organic pollution from the sample surface. Then, the original oxide layer on the sample surface was removed by soaking it in buffered oxide etch (BOE) solution for 3 min. In the BOE solution, the volume ratio of HF, NH_4_F, and deionized water was 1:10:50. The samples were immediately cleaned in a 270 kHz ultrasonic cleaner with deionized water for 5 min after the oxide layer had been removed. Transfer samples from which the oxide layer had been removed, was added to various CMMRF solutions and soaked for 24 h at 26 °C. We then took out the samples and cleaned them for 5 min in a 270 kHz ultrasonic cleaner with deionized water. The Young’s modulus of the modified layer was measured with a Bruker Dimension Icon AFM equipped with a PeakForce QNM module, and the probe model used for the measurement was DNISP-HS. The measurement area was 5 μm × 5 μm, and the average of the three measurements was regarded as the Young’s modulus of the modified layer. FTIR (Nicolet iS20, Thermo Scientific, Waltham, MA, USA) was employed to detect the chemical composition of the modified layer.

## 3. Results and Discussion

The design idea of the CMMRF fluid is based on the uniform wet etching technique for single-crystal silicon. First, a powerful oxidant is chosen to uniformly oxidize the surface, followed by the removal of the oxide layer to obtain an ultra-smooth surface.

The Fenton reagent contains a large amount of ·OH, and the oxidation potential of ·OH has reached 2.80 V, which is suitable for oxidizing the single-crystal silicon. Meanwhile, the concentration of Fe^3+^ or Fe^2+^ in the homogenous Fenton reagent is too high for direct discharge without treatment.

In the heterogeneous Fenton reagent, H_2_O_2_ reacts on the surface of the solid catalyst to generate ·OH, and the chemical reaction is as follows [36]:

(1) $$\equiv Fe^{3+}+H_{2}O_{2}\to Fe^{2+}+HO_{2}\cdot +H^{+}$$

(2) $$\equiv Fe^{2+}+H_{2}O_{2}\to Fe^{3+}+\cdot OH+OH^{-}$$

where the ≡Fe^2+^ and ≡Fe^3+^ represent the iron ions on the solid catalyst surface, and Fe^2+^ and Fe^3+^ represent the iron ions in the solution. In an acidic environment, redox cycles occur on the catalyst surface and in the liquid. Ferrihydrite [37], goethite [38], and Fe_3_O_4_ [39] are frequently utilized in heterogeneous Fenton reagents. These catalysts may be separated and reused through magnetic suction or sedimentation filtering, effectively preventing environmental contamination. Nano-Fe_3_O_4_ has exceptional catalytic and magnetic properties, and is widely utilized in MRF fluid and heterogeneous Fenton reagent. In this article, nano-Fe_3_O_4_ is used to develop a novel CMMRF fluid.

There are three factors affecting the oxidation effect of heterogeneous Fenton reagent, namely, pH value, concentration of H_2_O_2_, and concentration of the catalyst [40]. The concentration of ≡Fe^2+^ and ≡Fe^3+^ on the surface of the catalyst is the primary reason why pH influences oxidation activity. As the pH increases, the metal ions will precipitate, the oxidation potential of ·OH will decrease at the same time, and H_2_O_2_ is easily degraded into oxygen and water, which influences the generation of ·OH. For the concentration of H_2_O_2_, when H_2_O_2_ is inadequate, the amount of generated ·OH will be insufficient, whereas an excess of H_2_O_2_ will lead to the annihilation of ·OH.

Within a certain concentration range, the active site increases with the concentration of the catalyst, but excessive catalyst will consume the generated ·OH. The reaction is shown in the following equation:

(3) $$\equiv Fe^{2+}+\cdot OH\to Fe^{3+}+OH^{-}$$

To optimize these three factors, the absorbance of the mixture of CMMRF fluid and ·OH catcher was measured.

**Figure 2:**
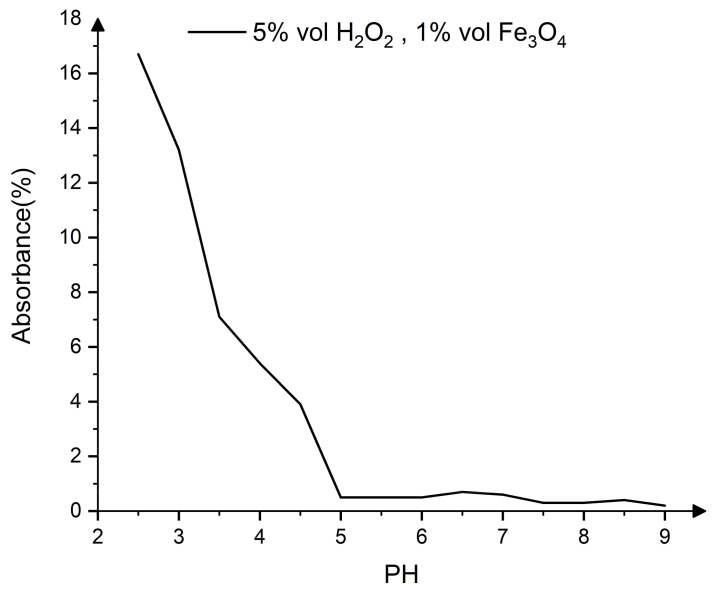
Effect of pH on absorbance.

As pH regulators, CH_3_COOH and NaOH are utilized, and the range of pH is between 2.5 and 9. As shown in the Figure 2, the reagent absorbance and the pH value have an obvious negative correlation. When the pH is greater than 6, the absorbance of the reagent is close to 0, which shows that the reagent contains almost no ·OH.

CH_3_COOH cannot be totally ionized since it is a monobasic weak acid. When pH = 2.5, the concentration of CH_3_COOH is 0.5 mol/L (1.5% vol), and when pH = 3 it is 0.05 mol/L (0.15% vol). The absorbance of the reagent is only 3.5 higher at pH = 2.5 than at pH = 3. Therefore, the pH of CMMRF fluid is set to 3, which saves CH_3_COOH, prevents the corrosion of processing equipment caused by high CH_3_COOH concentrations, and ensures the oxidation effect of CMMRF fluid.

**Figure 3:**
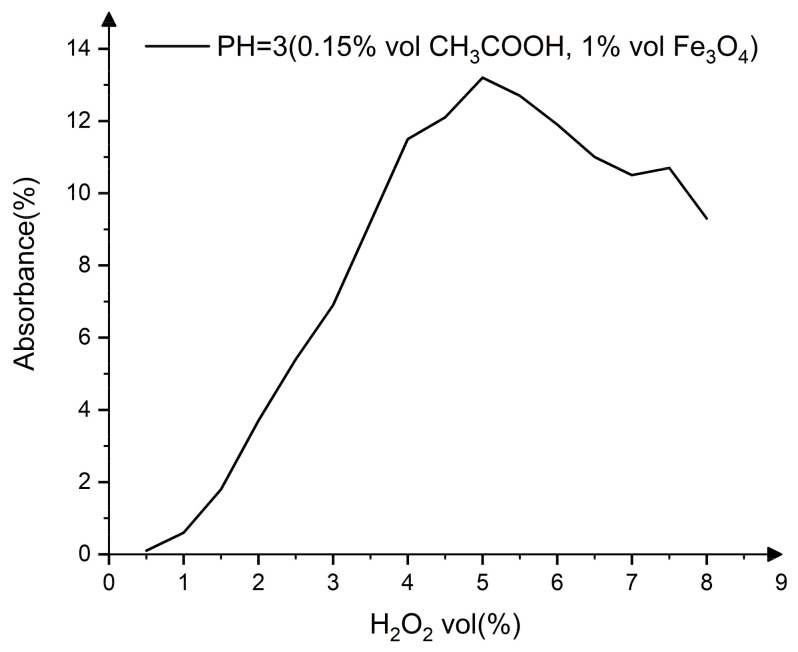
Effect of H_2_O_2_ concentration on absorbance.

The effect of H_2_O_2_ concentration on absorbance is shown in Figure 3. As the concentration of H_2_O_2_ rises, the absorbance of the reagent progressively increases, reaching a peak value at 5% vol H_2_O_2_, and then decreases as the concentration of H_2_O_2_ continues to rise.

**Figure 4:**
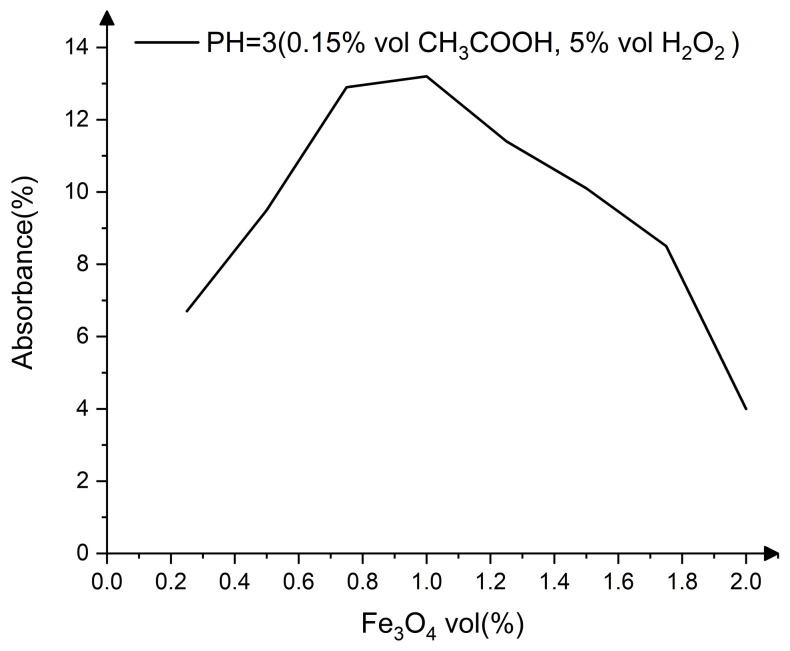
Effect of Fe_3_O_4_ concentration on absorbance.

The effect of Fe_3_O_4_ on absorbance follows the same pattern as that of H_2_O_2_, with an initial increase followed by a decline. Figure 4 shows that when the volume of Fe_3_O_4_ is 1%, the reagent produces the maximum concentration of ·OH.

The optimized CMMRF fluid composition is deionized water 60% vol, H_2_O_2_ 5% vol, CH_3_COOH 0.15% vol, CIP 25% vol, nano-Fe_3_O_4_ 1% vol, nanodiamond abrasive 3% vol, and PEG200 5.85% vol. Nanodiamond abrasive consists of carbon atoms, and possesses ultra-high hardness and stable chemical characteristics at room temperature. H_2_O_2_ is frequently used for disinfection and sterilization at low concentrations. CH_3_COOH is an organic acid that is the primary component of vinegar. A suitable amount of CIP can be used as a dietary additive to supplement the needs of the human body. PEG200 is polyethylene glycol with an average molecular weight of 200. It is a green organic solvent that can distribute particles in CMMRF fluid and adjust its viscosity. It is evident that the components of the CMMRF fluid are harmless to the environment and operators.

**Figure 5:**
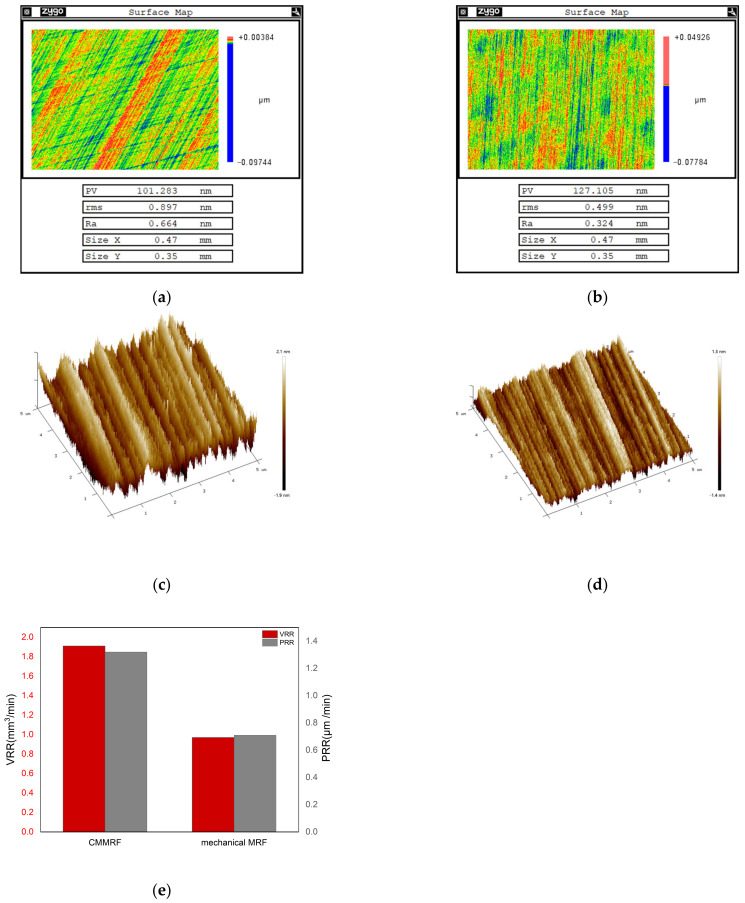
Polishing results of different MRF fluid:(**a**) Surface roughness after mechanical MRF process (measured by WLI); (**b**) surface roughness after CMMRF process (measured by WLI); (**c**) surface roughness after mechanical MRF process (measured by AFM); (**d**) surface roughness after CMMRF process (measured by AFM); (**e**) comparison of material removal rate of mechanical MRF and CMMRF.

As a comparison of CMMRF fluid, the MRF fluid with pure mechanical material removal is configured as follows: deionized water 65% vol, CIP25% vol, nanodiamond 3%, PEG200 7% vol, and pH adjusted to 3 with HCl, which is consistent with the CMMRF fluid. Comparing the performance of these two kinds of fluid, Figure 5 shows the results of surface roughness Ra and VRR after different MRF fluid processing. From the surface roughness results measured by WLI and AFM, it can be seen that the surface texture after CMMRF is more uniform and the surface quality after CMMRF is also significantly improved. Furthermore, the VRR of the novel CMMRF fluid is 1.91 × 10^−2^ mm^3^/min, which increases by 196.9% compared with 0.97 × 10^−2^ mm^3^/min obtained by the mechanical MRF fluid. The peak removal rate (PRR) of CMMRF is 1.32 μm/min, which is 186.6% greater than the PRR of the mechanical MRF fluid (0.71 μm/min). Therefore, it is demonstrated that the novel green CMMRF fluid has superior performances when polishing single-crystal silicon.

**Figure 6:**
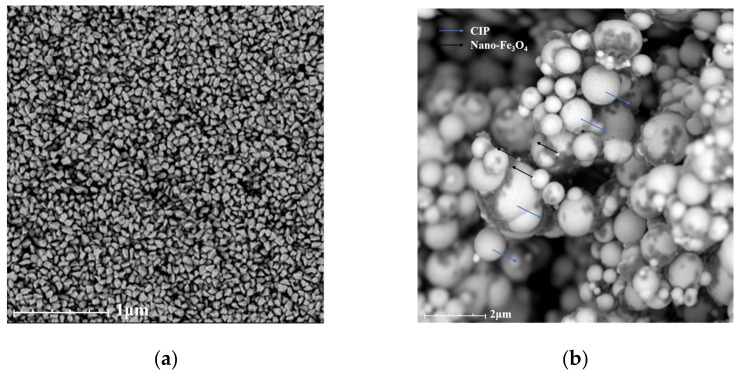
(**a**) SEM image of nanodiamond abrasive; (**b**) CIP mixed with nano-Fe_3_O_4_.

To characterize the performance of the CMMRF fluid, the nanodiamond abrasive and CIP mixed with nano-Fe_3_O_4_ were photographed by a SEM in the CMMRF base fluid environment. It can be seen in Figure 6a that the nanodiamond has a good dispersion and homogeneous particle size, which is beneficial to the processing of a high-quality surface. The shape of the nanodiamond is an irregular polygon, and each side of the polygon can be regarded as a sharp micro-cutting edge, which is helpful to improve the material removal efficiency. In Figure 6b, it can be seen that both CIP and nano-Fe_3_O_4_ are spherical, and the regular spherical shape is beneficial for preventing surface defects during processing.

**Figure 7:**
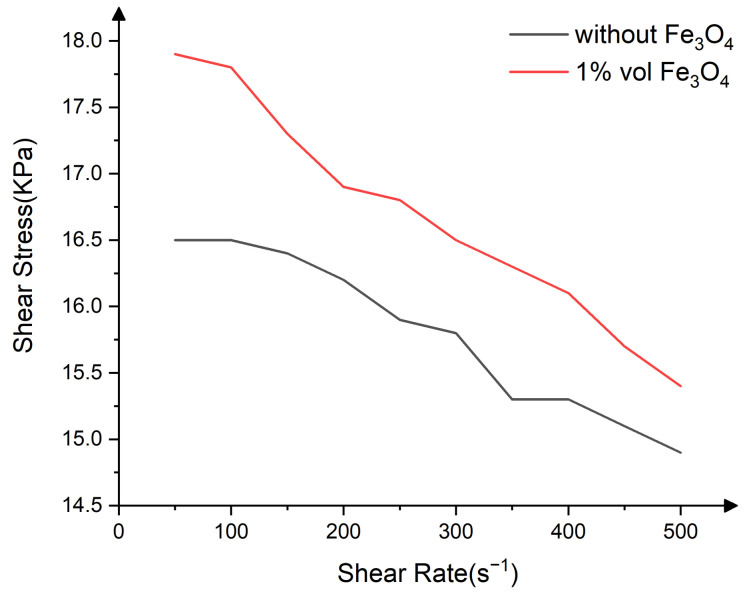
The magnetic rheometer test results of different MRF fluid.

Figure 7 shows the test results of the magnetic rheometer. The shear yield strength of MRF fluid increased by 5.7% at 250 s^−1^ when nano-Fe_3_O_4_ was added. Under the influence of an external directional magnetic field, the CIPs are arranged in a chain structure, and nano-Fe_3_O_4_ is embedded in the gap between the CIP and enhances the magnetic dipole–dipole force, which is reflected as an increase of shear yield strength. With an increase in shear yield strength, the capacity of MRF fluid to hold nanodiamond polishing particles is enhanced, which is beneficial for improving the removal efficiency of materials.

To investigate the reaction mechanism of CMMRF on the single-crystal silicon, various chemical substances were added to configure the different MRF fluids, and three samples with its original oxide layer removed were immersed in it. As shown in Table 2, fluid No. 1 and No. 2 are adjusted to pH = 3 with HCl, while fluid No. 3 is adjusted to pH = 3 using CH_3_COOH.

**Figure 8:**
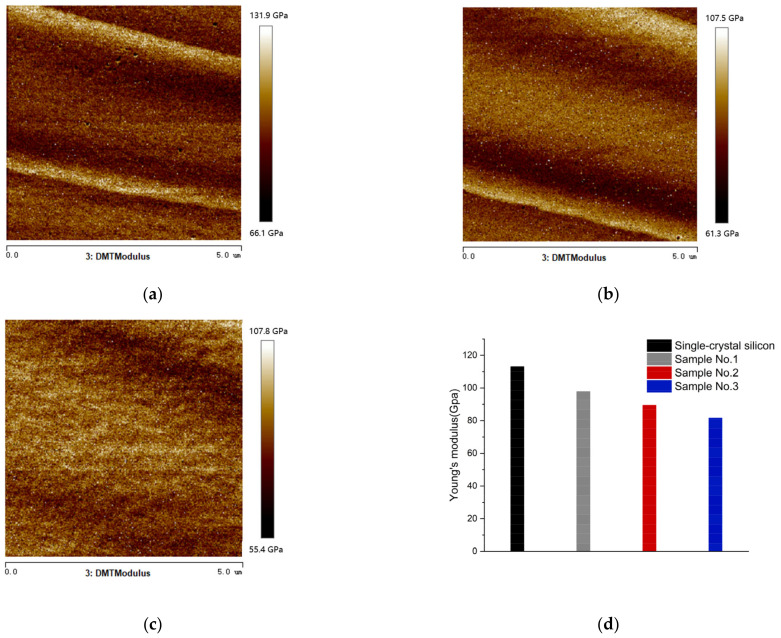
Young’s modulus test results: The Young’s modulus image of sample (**a**) No. 1; (**b**) No. 2; (**c**) No. 3; (**d**) average values of Young’s modulus of different samples.

Young’s modulus is measured at three points on each sample, with a range of 5 μm × 5 μm at each point. The average of three points is regarded as the Young’s modulus of this sample. The measurement results are shown in Figure 8. The Young’s modulus of single-crystal silicon is 113 GPa [41]. As shown in Figure 8, after 24 h of immersion in MRF fluid, the Young’s modulus of the single-crystal silicon sample decreased to 97.8 GPa, and the Young’s modulus of Sample No. 2 and Sample No. 3 decreased to 89.5 GPa and 81.6 GPa, respectively.

**Figure 9:**
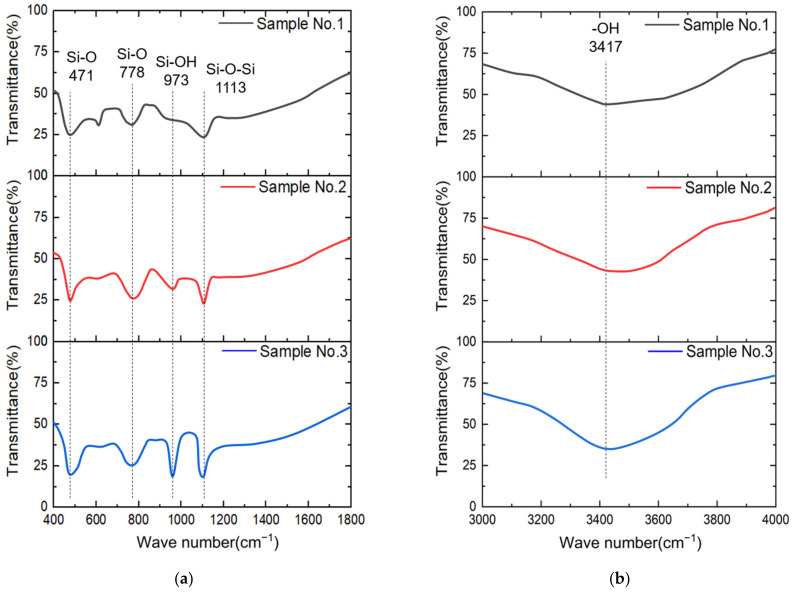
FTIR spectra of different samples: (**a**) 400–1800 cm^−1^; (**b**) 3000–4000 cm^−1^.

From Figure 9a, it can be seen that each sample has strong peaks at the position of 471 cm^−1^ (Si-O), 778 cm^−1^ (Si-O), and 1113 cm^−1^ (Si-O-Si) [42], which are the characteristic peaks of SiO_2_, and the absorption peaks of Samples No. 2 and No. 3 are significantly stronger than that of Sample No. 1. In addition, compared with Sample No. 1 and No. 2, Sample No. 3 has a strong absorption band at 973 cm^−1^ (Si-OH). It can be seen from Figure 9b, at the position of 3417 cm^−1^ (OH group), the absorption bands of Sample No. 1, Sample No. 2, and Sample No. 3 are intensified sequentially [43].

During the Young’s modulus test, the degradation of the sample mechanical properties will be aggravated by a thicker modified layer. It can be inferred that the thickness of the modified layer of Sample No. 1 is less than that of other samples, and the absorption peak intensity of Sample No. 2 and Sample No. 3 is higher than that of Sample No. 1, confirming this conclusion again. This is because the heterogeneous Fenton reagent composed of nano-Fe_3_O_4_ and H_2_O_2_ has a better oxidation performance and generates a thicker modified layer within the same treatment time.

The modified layer of Sample No. 3 is attacked by the hydroxyl group in CH_3_COOH, which destroys the structure of the oxidation product SiO_2_, resulting in a further decrease in Young’s modulus [44,45,46]. According to the research, the VRR of MRF is proportional to the Young’s modulus; hence, the soft modified layer leads to a significant increase in MRF removal efficiency.

**Figure 10:**
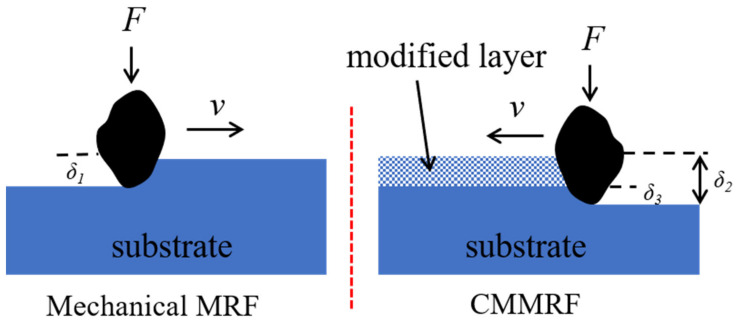
Abrasive contact model of mechanical MRF and CMMRF.

As depicted in Figure 10, the nondiamond abrasive entirely removed the modified layer in the CMMRF process. The presence of the modified layer reduces the substrate penetration depth (δ_3_) of abrasives, while the total penetration depth (δ_2_) is greater than that of mechanical MRF (δ_1_). The microscopic topography formed by abrasives will be more uniform when the substrate penetration depth is lower, and an increase in the total penetration depth will improve the material removal efficiency.

Furthermore, it should be noted that the chemical-mechanical balance and electrostatic force between the abrasive and modified layer also play vital roles in the high-efficiency ultra-smooth process. Further research is needed to fill the gaps in the CMMRF material removal mechanism.

## 4. Conclusions

In this paper, a novel CMMRF fluid is developed and realizes high-efficiency ultra-smooth processing of single-crystal silicon. The novel fluid is harmless to the environment and the operators, and the solid components in the fluid can be recycled and reused.

The polishing mechanism of CMMRF was revealed by comprehensive characterizations. The nano-Fe_3_O_4_ enhanced the shear yield strength of the fluid, and the employment of the hard nanodiamond abrasive was beneficial for improving the material removal efficiency. The heterogeneous Fenton reagent is composed of Fe_3_O_4_ and H_2_O_2_; the ·OH can rapidly oxidize single-crystal silicon. The addition of CH_3_COOH considerably enhanced the material removal rate by destroying the modified layer with the hydrolysis reaction. In addition, the modified layer reduces the substrate penetration depth of the abrasives, resulting in a significant improvement in surface roughness. This article could be a reference for the high-efficiency ultra-smooth processing of silicon. After the optimization of processing parameters, it can also be applied to a variety of materials as far as the modified layer can be generated on the surface.

## Figures and Tables

**Table 1:** Constituents used for configuring MRF fluid.

Constituents	Purity	Supplier
CIP	-	BASF, Ludwigshafen, Germany
Nano-Fe_3_O_4_	-	Delta, Xiamen, China
Diamond abrasive	-	Huanghe Whirlwind, Zhengzhou, China
H_2_O_2_	AR	Sinopharm Chemical Reagent, Shanghai, China
CH_3_COOH	AR	Sinopharm Chemical Reagent, Shanghai, China
PEG200	AR	OKA, Beijing, China
HCl	AR	Sinopharm Chemical Reagent, Shanghai, China
NaOH	AR	Sinopharm Chemical Reagent, Shanghai, China

**Table 2:** Components of different fluid.

Number	H_2_O_2_ (vol%)	Fe_3_O_4_ (vol%)	CH_3_COOH (vol%)	pH
1	5	-	-	3
2	5	1	-	3
3	5	1	0.15	3

## Data Availability

Data are contained within the article.
